# Integrating Genetics With Epidemiological Measurements Identifies Burden QTLs of Inflammatory Bowel Disease across 20 Countries

**DOI:** 10.1002/advs.202519007

**Published:** 2026-09-27

**Authors:** Chen Sun, Siyu Wei, Junxian Tao, Haiyan Chen, Chi Yan, Jiacheng Wang, Jing Xu, Lian Duan, Yuanbo Zhan, Yuping Zou, Ruilin Li, Linna Yuan, Wei She, Songlin Lu, Ning Wang, Qinduo Ren, Yan Guo, Yuan Xu, Chang Wang, Hongsheng Tian, Chen Zhang, Yu Dong, Guoping Tang, Zhenwei Shang, Yongshuai Jiang, Wenhua Lv, Mingming Zhang, Hongchao Lyu

**Affiliations:** ^1^ College of Bioinformatics Science and Technology Harbin Medical University Harbin China; ^2^ Faculty of Information Engineering and Automation Kunming University of Science and Technology Kunming China; ^3^ Central Laboratory The First Affiliated Hospital of Wenzhou Medical University Wenzhou China; ^4^ Department of Periodontology and Oral Mucosa The Second Affiliated Hospital of Harbin Medical University Harbin China; ^5^ Harbin Medical University Harbin China; ^6^ The Fourth Affiliated Hospital Zhejiang University School of Medicine Yiwu China

**Keywords:** burden quantitative trait locus, epidemiological, genetics, inflammatory bowel disease

## Abstract

It remains unclear whether the observed heterogeneity in the burden of inflammatory bowel disease (IBD) across countries is associated with differences in population genetic structure. To address this problem, we performed a burden quantitative trait locus (bQTL) analysis, integrating epidemiological data (1990–2021) from the Global Burden of Disease (GBD) study with genotypes of 2 621 978 SNPs from 2414 unrelated individuals in the 1000 Genomes Project across 20 matched countries. We identified 1074 bQTLs of IBD, of which 384 were significantly associated with both the age‐standardized prevalence rate and age‐standardized incidence rate. rs7633471 (chr3:30698616:C>A) showed the strongest effect: each increase in centi‐allele frequency (CAF) of the allele C was associated with a 10.00 decrease in age‐standardized prevalence rate and a 1.02 decrease in age‐standardized incidence rate. Pathway analysis revealed that bQTLs were primarily enriched in synaptic membrane, voltage‐gated potassium channel complex, and potassium channel complex. Through fine‐mapping, we further identified 800 putative causal variants for IBD, with significant findings located within genes related to the immune system (*IGSF21* and *CSMD2*), inflammatory response (*SMPDL3B* and *AOAH*), and intestinal cancers (*FHIT* and *CSMD2*). Our study provides the first comprehensive characterization of the genetic architecture underlying the global burden of IBD.

## Graphical abstract

The burden quantitative trait locus (bQTL) analysis integrates epidemiological data from GBD study with genotype of 2 621 978 SNPs from 2414 individuals in 1000 Genomes Project across 20 countries. rs7633471 shows the strongest effect that each increase in centi‐allele frequency of the allele C associated with 10.00 decrease of age‐standardized prevalence rate and 1.02 decrease of age‐standardized incidence rate.

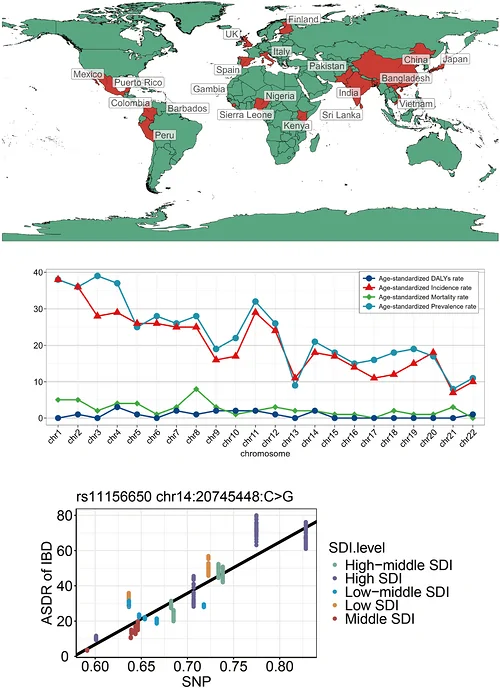

## 1 Introduction

Inflammatory bowel disease (IBD), encompassing Crohn's disease (CD) and ulcerative colitis (UC), is a chronic, debilitating disorder of the gastrointestinal tract with rising global incidence, particularly in newly industrialized regions [1, 2, 3, 4, 5, 6]. The disease often requires lifelong treatment and leads to complications such as strictures, fistulae, and colorectal cancer, imposing a substantial healthcare and economic burden [7, 8, 9, 10, 11, 12].

In 2021, the age‐standardized incidence rate of IBD in United Kingdom was 15.94 per 100 000 (95%UI, 13.95–18.38), almost 2 times than Italy (8.25 per 100,000, 95%UI, 7.17–9.71), 2.6 times more than Pakistan (6.04 per 100 000, 95%UI, 5.22–7.30), and nearly 80 times more than Mexico (0.20 per 100,000, 95%UI, 0.17–0.25), highlighting the high heterogeneity of the burden of IBD in different countries. Currently, a great number of studies tend to focus on socioeconomic and environmental factors, such as income level, age, and sex, to assess disease burden [13, 14, 15, 16, 17]. Lin et al. documented that the age‐standardized incidence rate increased the most in upper‐middle‐income regions, and no significant gender disparities in disease burden were observed [3]. However, genetic factors, which play a significant role in disease etiology and population health outcomes and demonstrate distinct regional heterogeneity, are rarely reported in the current research on disease burden. A growing body of evidence underscores the heterogeneity of the effects of genetic loci among populations [18, 19, 20, 21]. For example, Z liu et al. demonstrated that major European risk variants in *NOD2* and *IL23R* of IBD are not present in individuals of East Asian ancestry [22]. Is there a link between cross‐country variations in disease burden of IBD and genetic architecture? There is still no study that has examined the association between the two.

**FIGURE 1:**
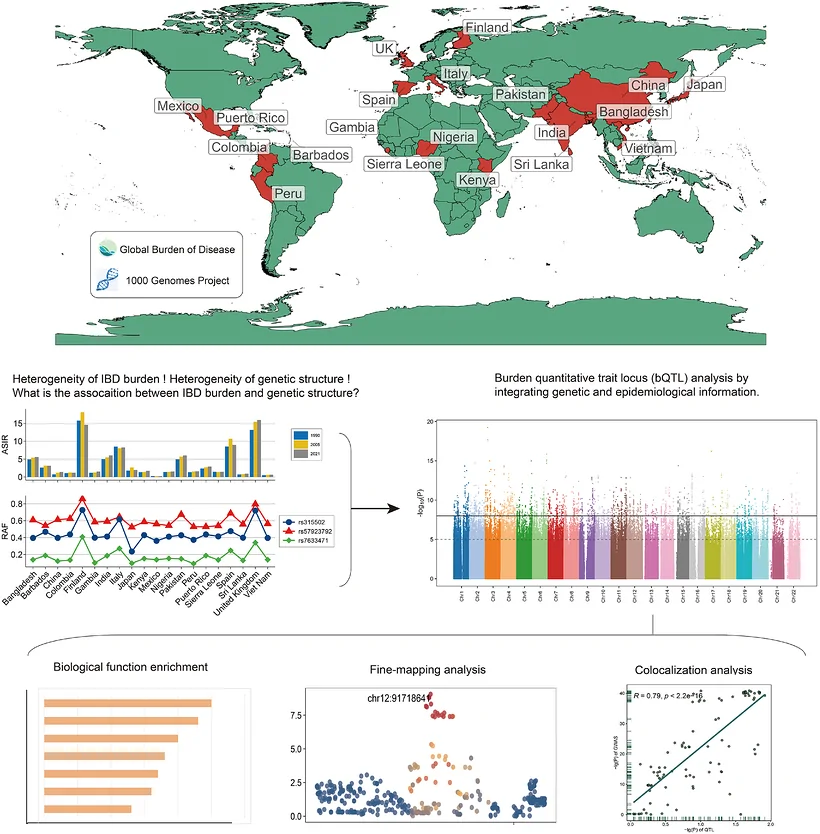
Study overview.

To solve the problem, we integrated genotypes of 2 621 978 SNPs for 2414 unrelated individuals from the 1000 Genomes Project and epidemiological information from 1990 to 2021 in the Global Burden of Disease (GBD) study across 20 matched countries to dissect genetic associations with the global burden of IBD. Our study aimed to 1) identify burden quantitative trait loci (bQTL) of IBD; 2) uncover biological pathways linked to bQTLs; 3) perform fine mapping to prioritize causal variants; and 4) investigate the relationships between bQTLs and genetic associations identified by traditional GWAS (Figure 1).

## 2 Result

### 2.1 High Heterogeneity of Inflammatory Bowel Disease Burden Among Countries

Table 1 reports the age‐standardized incidence, prevalence, mortality, and Disability‐Adjusted Life Years (DALY) for IBD in 20 countries (Barbados, Gambia, Nigeria, Kenya, Sierra Leone, China, Vietnam, Japan, Finland, United Kingdom, Italy, Spain, Pakistan, Bangladesh, India, Sri Lanka, Puerto Rico, Colombia, Peru, and Mexico) as a whole from 1990 to 2021 (every five years, part S1). Detailed information on annual age‐standardized estimates in each country is reported in Table S1.

**TABLE 1:** Age‐standardized rates (per 100,000 population) of prevalence, incidence, mortality, and disability‐adjusted life years (DALY) attributable to inflammatory bowel disease in 20 countries.

	Prevalence	Incidence	Mortality	DALY
1990	25.62[23.34,27.85]	2.91[2.66,3.17]	0.59[0.48,0.69]	18.59[16.03,21.11]
1995	26.97[24.64,29.01]	3.11[2.83,3.39]	0.56[0.48,0.66]	18.23[15.95,20.52]
2000	28.45[25.67,31.10]	3.41[3.10,3.72]	0.53[0.45,0.62]	17.56[15.34,19.67]
2005	29.28[26.65,31.83]	3.47[3.16,3.79]	0.53[0.45,0.60]	17.06[15.12,19.02]
2010	32.61[29.61,35.73]	3.90[3.55,4.26]	0.45[0.40,0.50]	15.94[14.36,17.53]
2015	32.74[29.59,35.93]	3.94[3.56,4.32]	0.40[0.35,0.45]	14.72[13.11,16.34]
2020	30.92[27.88,33.88]	3.80[3.45,4.16]	0.37[0.32,0.43]	13.66[12.04,15.25]
2021	30.82[27.74,33.81]	3.80[3.44,4.17]	0.37[0.31,0.42]	13.60[11.92,15.27]
Percent change between 1990 and 2021	20.30%	30.58%	−37.29%	−26.84%

**FIGURE 2:**
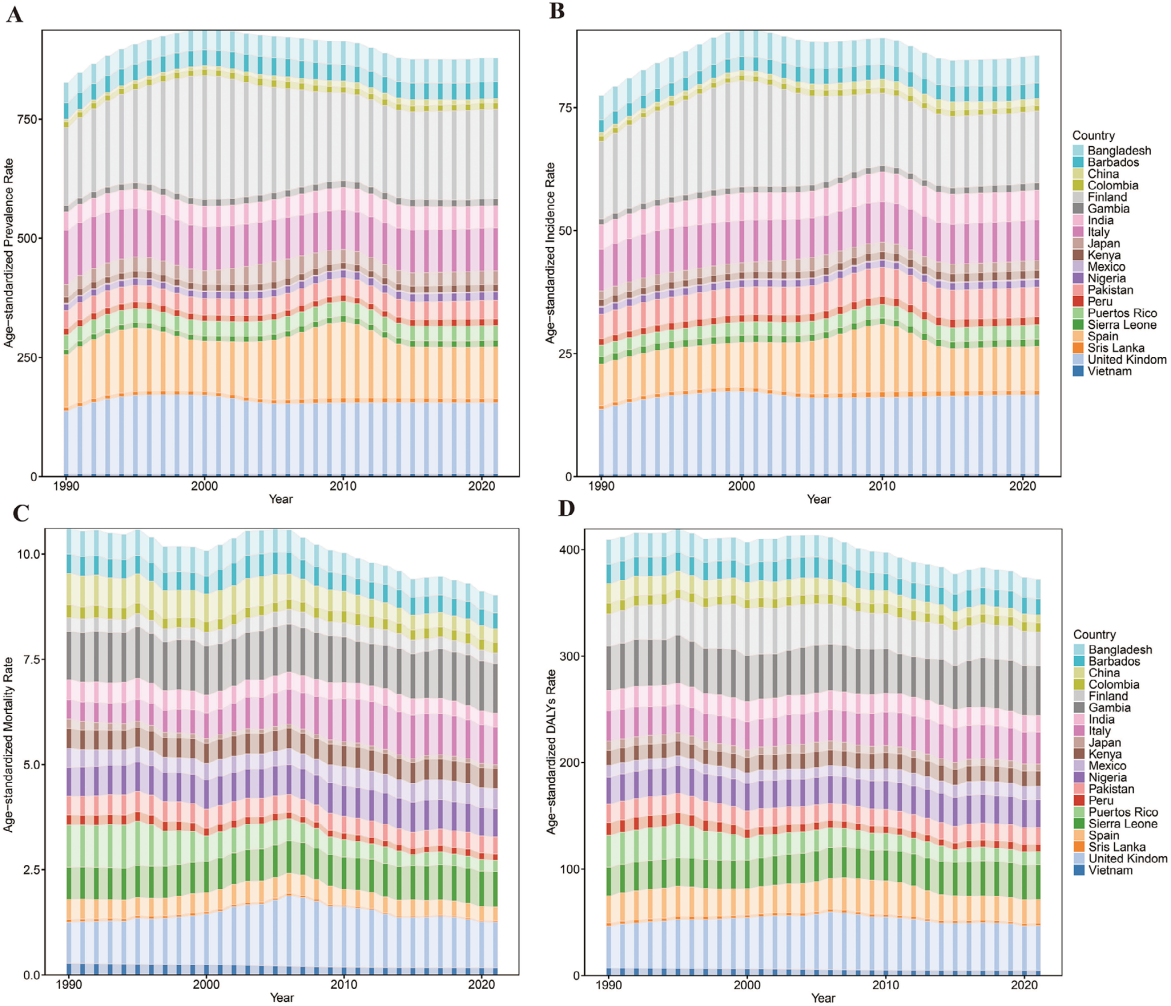
Heterogeneity of IBD burden in 20 countries. (A) Age‐standardized prevalence rate, (B) age‐standardized incidence rate, (C) age‐standardized mortality rate, and (D) age‐standardized DALY rate in each country from 1990 to 2021.

In 2021, over 1.3 million people were affected by IBD in 20 countries, which yields an age‐standardized prevalence rate of 30.82 per 100 000 (95%UI = 27.74–33.81). Among them, Finland, the United Kingdom, and Spain showed the highest age‐standardized prevalence rate with more than 100 per 100 000 population, while Mexico, Vietnam, Sri Lanka, and China were less than 10 per 100 000, representing a more than 10‐fold disparity (Figure 2A). Incident cases were almost 160 000 in 20 countries, yielding an age‐standardized incidence rate of 3.80 per 100 000 (95% UI = 3.44–4.17). The highest age‐standardized incidence rate was observed in the United Kingdom (15.94 per 100,000, 95% UI = 13.95–18.38), while Mexico displayed the lowest (0.20 per 100,000, 95% UI = 0.17–0.25), being 80 times smaller than the United Kingdom (Figure 2B). Moreover, age‐standardized mortality rates in Gambia and the United Kingdom were more than 1 per 100 000, while in Sri Lanka and Japan they were less than 0.1 per 100 000 (Figure 2C). Age‐standardized DALY rates in Gambia and the United Kingdom were more than 40 per 100 000, while Sri Lanka was less than 4 per 100 000 (Figure 2D). It is obvious that the burden of IBD is highly heterogeneous across diverse countries.

### 2.2 Discovery of 1074 Burden QTLs for IBD

**FIGURE 3:**
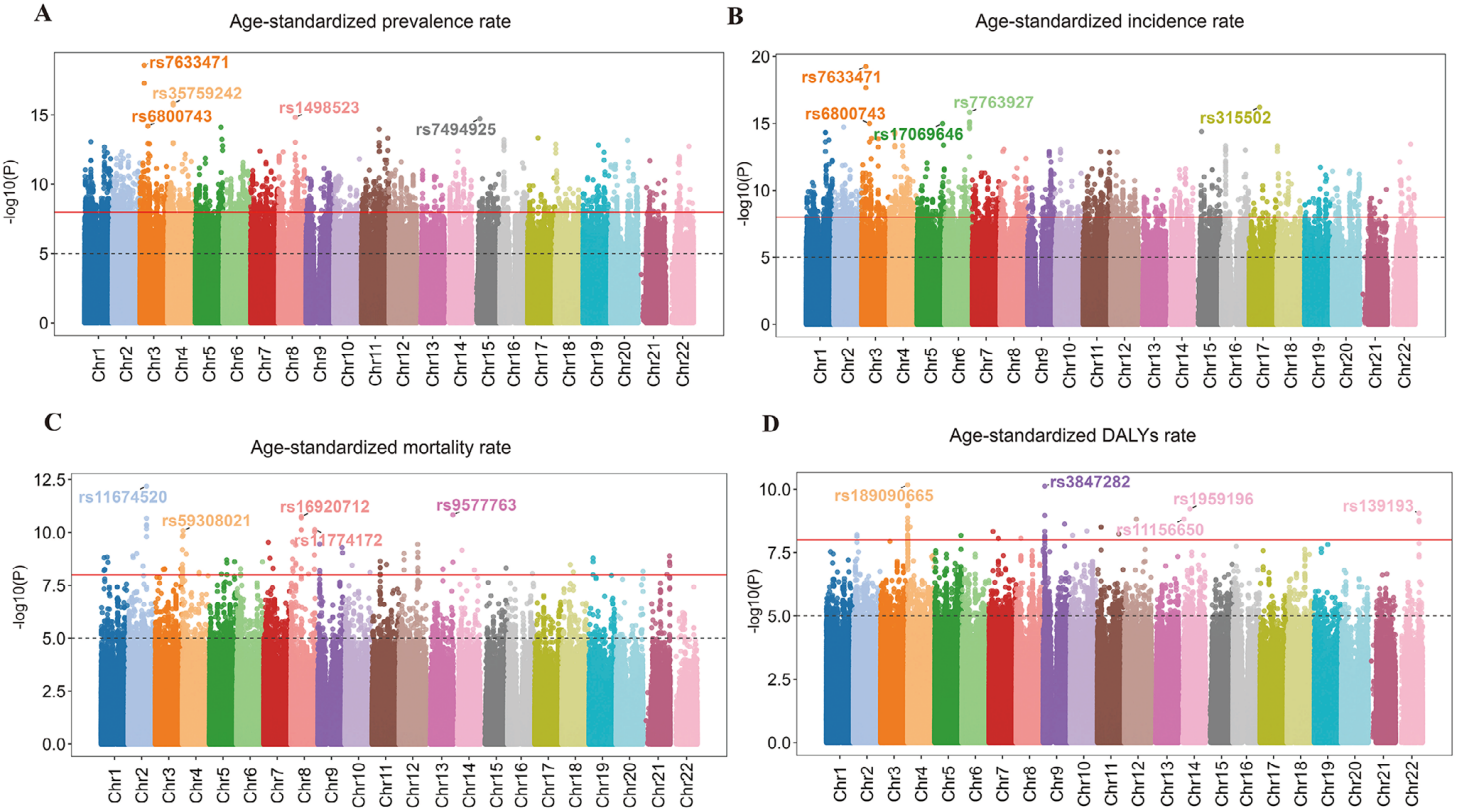
Manhattan plots identify bQTLs associated with IBD. bQTLs of (A) age‐standard prevalence rate, (B) age‐standard incidence rate, (C) age‐standard mortality rate, and (D) age‐standard DALY rate. The top five lead SNPs are labeled.

We conducted a burden quantitative trait locus (bQTL) analysis to identify genetic variants associated with the global burden of IBD. Out of the 2 621 978 single‐nucleotide polymorphisms (SNPs) analyzed, 774 lead SNPs demonstrated genome‐wide significant associations (*p* < 1 × 10^−^^8^) with age‐standardized prevalence rate (Figure 3A and Table S2). Among them, rs7633471 (chr3:30698616:C>A, beta_CAF_ = −10.00, *p* = 2.59 × 10^−19^) demonstrated the strongest association that each centi‐allele frequency (CAF) addition of the C allele, 10.00 decrease of age‐standardized prevalence rate of IBD was observed. The followed significant variants were rs35759242 (chr4:38211638:A>G, beta_CAF_ = 9.01, *p* = 1.42 × 10^−16^), rs1498523 (chr8:88782290:G>T, beta_CAF_ = ‐8.02, *p* = 1.52 × 10^−15^), rs7494925 (chr15:28844663:G>C, beta_CAF_ = ‐10.11, *p* = 1.92 × 10^−15^), and rs6800743 (chr3:56844919:T>C, beta_CAF_ = ‐9.01, *p* = 6.46 × 10^−15^) (Figure S1).

In the analysis of the age‐standardized incidence rate of IBD, 611 lead SNPs (Figure 3B and Table S3) met the criteria of conventional genome‐wide significance (*p* < 1 × 10^−8^). rs7633471 (chr3:30698616:C>A, beta_CAF_ = −1.02, *p* = 5.62 × 10^−20^) and rs6800743 (chr3:56844919:T>C, beta_CAF_ = −0.87, *p* = 1.01 × 10^−15^) were still the top 5 significant variants. Other noteworthy variants included rs315502 (chr17:31711581:T>G, beta_CAF_ = 0.77, *p* = 5.99 × 10^−17^), rs7763927 (chr6:153906454:T>C, beta_CAF_ = 0.87, *p* = 1.43 × 10^−16^), and rs17069646 (chr5:168025246:T>C, beta_CAF_ = 1.18, *p* = 1.02 × 10^−15^). Notably, rs315502 and rs17069646 were also significantly associated with the age‐standardized prevalence rate of IBD, while rs7763927 was not (Figure S2).

For the age‐standardized mortality rates of IBD, we identified 56 lead SNPs (Figure 3C and Table S4). The top five were rs11674520 (chr2:164924998:A>G, beta_CAF_ = ‐0.14, *p* = 6.60 × 10^−13^), rs9577763 (chr13:112454065:G>A, beta_CAF_ = ‐0.12, *p* = 1.49 × 10^−11^), rs16920712 (chr8:56528451:T>C, beta_CAF_ = ‐0.15, *p* = 1.77 × 10^−11^), rs11774172 (chr8:128213256:A>G, beta_CAF_ = 0.13, *p* = 7.35 × 10^−11^), and rs59308021 (chr4:4843361:C>T, beta_CAF_ = −0.13, *p* = 8.18 × 10^−11^). On the contrary, the five variants were not significantly associated with both the age‐standardized prevalence rate and age‐standardized incidence rate of IBD (Figure S3).

A total of 18 lead SNPs showed genome‐wide significant associations (*p* < 1 × 10^−8^) with the age‐standardized DALY rate of IBD (Figure 3D and Table S5). The most significant variant was rs189090665 (chr4:395762:T>C, beta_CAF_ = ‐3.83, *p* = 6.74 × 10^−11^). The followed top variants were rs3847282 (chr9:8269791:A>G, beta_CAF_ = 1.76, *p* = 7.62 × 10^−11^), rs1959196 (chr14:44288914:T>C, beta_CAF_ = 3.31, *p* = 5.92 × 10^−10^), rs139193 (chr22:44209456:G>C, beta_CAF_ = ‐1.67, *p* = 8.76 × 10^−10^), and rs11156650 (chr14:20745448:C>G, beta_CAF_ = 2.91, *p* = 1.53 × 10^−9^) (Figure S4). Among them, rs11156650 was also associated with the age‐standardized incidence rate of IBD, but not significant in both age‐standardized prevalence rate and age‐standardized mortality rate of IBD.

Of note, we found that 384 lead SNPs were significantly associated with both the age‐standardized prevalence rate and age‐standardized incidence rate of IBD (Figure S5), of which rs7633471, rs315502, and rs6800743 demonstrated the top 3 strongest effects. With a per centi‐allele frequency addition of the C allele of rs7633471 (chr3:30698616:C>A), a 10.00 decrease in the age‐standardized prevalence rate and a 1.02 decrease in the age‐standardized incidence rate can be observed. Each centi‐allele frequency addition of the T allele of rs315502 (chr17:31711581:T>G) was associated with a 7.72 increase in the age‐standardized prevalence rate and a 0.77 increase in the age‐standardized incidence rate. For each increase in the centi‐allele frequency of the T allele of rs6800743 (chr3:56844919:T>C), we observed a corresponding 9.01 decrease in the age‐standardized prevalence rate and a 0.87 decrease in the age‐standardized incidence rate. One variant, rs11156650 (chr14:20745448:C>G), was significant for both the age‐standardized incidence rate and age‐standardized DALY rate. With each increase in centi‐allele frequency of the C allele, a 1.05 increase in age‐standardized incidence rate and a 2.91 increase in age‐standardized DALY rate can be observed (Figure S5).

### 2.3 Biological Function of Burden QTLs for IBD

**FIGURE 4:**
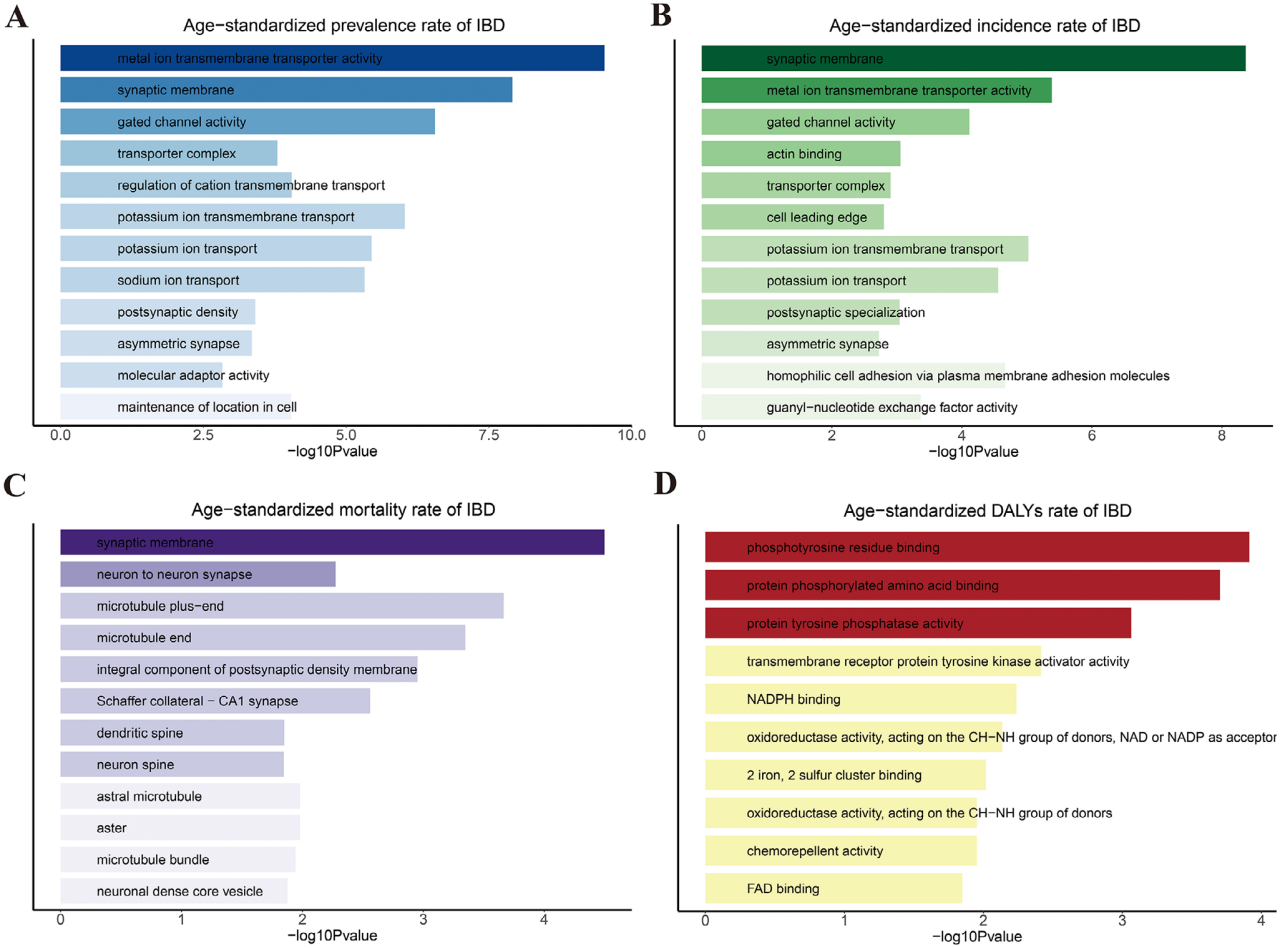
Biological functions of bQTLs associated with IBD. Top 12 enriched GO biological pathways of (A) age‐standard prevalence rate, (B) age‐standard incidence rate, (C) age‐standard mortality rate, and (D) age‐standard DALY rate.

To characterize the potential roles of the identified bQTLs of IBD, we performed functional enrichment analysis to investigate the biological properties and regulatory pathways (Figure 4 and Table S6). The bQTLs of both age‐standardized prevalence rate and age‐standardized incidence rate of IBD were enriched for some Gene Ontology (GO) terms, including metal ion transmembrane transporter activity, synaptic membrane, gated channel activity, transporter complex, potassium ion transmembrane transport, and so on (Figure 4A,B). Consistently, pathway analysis revealed that enrichment was mainly related to the digestive system (salivary and pancreatic secretion) and nervous system (cholinergic, dopaminergic, glutamatergic synapses, retrograde endocannabinoid signaling, and long‐term potentiation). Additional important pathways included purine metabolism, adrenergic signaling in cardiomyocytes, endocrine and other factor‐regulated calcium reabsorption, cAMP signaling pathway, etc. It highlighted the potential involvement of our bQTLs in diverse organismal systems and metabolic processes. Similarly, bQTLs associated with age‐standardized mortality rate of IBD were enriched for synaptic components, such as synaptic membrane, neuron‐to‐neuron synapse, and neuron spine (Figure 4C). Additional enriched GO terms were involved in microtubule plus‐end, astral microtubule, microtubule bundle, etc. The DALY rate‐associated bQTLs were enriched for phosphotyrosine residue binding, protein phosphorylated amino acid binding, and protein tyrosine phosphatase activity (Figure 4D).

### 2.4 Fine Mapping Identified 800 Putative Causal Variants of the Burden of IBD

We used the approximate Bayesian fine‐mapping (ABF) method to calculate posterior inclusion probability (PIP) for candidate bQTLs that met the genome‐wide significance. For the independent lead SNPs, a total of 800 loci were successfully fine‐mapped, including 508 in age‐standardized prevalence rate analysis, 452 in age‐standardized incidence rate analysis, 54 in age‐standardized mortality rate analysis, and 18 in age‐standardized DALY rate analysis (Table S7). Notably, 232 loci showed pleiotropy, affecting both age‐standardized prevalence rate and age‐standardized incidence rate (Figure S6). Among them, 8 genes were found to overlap with previously reported IBD‐associated genes, including *ANK3*, *BANK1*, *DOCK2*, *IL21‐AS1*, *KIAA0319*, *SGIP1*, *NELL1*, and *SLC35D1*. Except for *IL21‐AS1*, an lncRNA, all are protein‐coding genes. Functional annotation revealed that these overlapping genes are predominantly involved in clathrin‐mediated vesicle trafficking and endocytosis, specialized neuromuscular structures, and cytoskeletal anchoring required for lymphocyte migration, as well as molecular adaptor activities and nucleotide‐sugar transport. The functions highlighted their roles in membrane dynamics, signal transduction, and muscle/nerve function (Figure S18). Notably, mutations in *DOCK2* result in immunodeficiency 40 (IMD40), a combined form of immunodeficiency that affects T cell number and function, underscoring the immunological relevance of these loci.

Of particular interest, we found that some pleiotropic putative causal variants are located within immunoglobulin‐related genes. A notable example is *IGSF21* (*immunoglobulin superfamily member 21*, rs9659886 [chr1:18202617:T>A]), which encodes an immunoglobulin superfamily member containing two immunoglobulin (Ig) domains [https://www.ncbi.nlm.nih.gov/gene/84966]. Similarly, we identified a variant in *LSAMP* (*limbic system associated membrane protein*, rs2048971 [chr3:116666626:T>C]) which encodes a member of the immunoglobulin LAMP and may mediate neural‐immune interactions relevant to IBD [https://www.ncbi.nlm.nih.gov/gene/4045]. The other noteworthy gene, *CAMTA1* (*Calmodulin‐binding transcriptional activator 1*, rs10864254 [chr1:6954047:T>C] and rs705676 [chr1:7350003:T>C]), encodes proteins containing a transcription factor immunoglobulin domain [https://www.ncbi.nlm.nih.gov/gene/23261] and plays important roles in cell cycle regulation, cell differentiation, initial development, and maturation [23]. In addition, our study implicated two genes with established roles in intestinal cancers. *FHIT* (*fragile histidine triad diadenosine triphosphatase*, rs6779703 [chr3:60048394:G>T]) showed aberrant transcripts in colon carcinomas [https://www.ncbi.nlm.nih.gov/gene/2272]. *CSMD2* (*CUB and Sushi multiple domains 2*, rs12755908 [chr1:34151655:C>T]) encodes proteins involved in the control of the complement cascade of the immune system and may act as a tumor suppressor for colorectal cancer [https://www.ncbi.nlm.nih.gov/gene/114784].

For the analysis of putative causal SNPs of age‐standardized mortality rate, we found two protein‐coding genes involved in the inflammatory response. *SMPDL3B* (*sphingomyelin phosphodiesterase acid like 3B*, rs4644519 [chr1:27956760:G>A]) was predicted to be involved in negative regulation of the inflammatory response and is biasedly expressed in the colon [https://www.ncbi.nlm.nih.gov/gene/27293]. *AOAH* (*acyloxyacyl hydrolase*, rs7797451 [chr7:36667363:C>T]) encodes a protein that plays a role in modulating host inflammatory response to gram‐negative bacteria [https://www.ncbi.nlm.nih.gov/gene/313].

### 2.5 Colocalization Analysis Between Burden QTLs and Conventional GWAS of IBD

To explore the relations between burden QTLs and conventional genome‐wide association study (GWAS) of IBD, we conducted a colocalization analysis with a GWAS meta‐analysis [22]. We defined genomic clumps (linkage disequilibrium threshold r^2^> 0.1 and a region of ± 250 kb around lead SNPs) and observed similar significance patterns between bQTL analysis and GWAS in several clumps.

Across 75 clumps identified, 18 clumps showed moderate correlation (Pearson correlation coefficient R > 0.5 and *p* < 0.05) with GWAS loci. A notable example is the clump of lead SNP rs7608429 (chr2:68353704:G>T, Pearson correlation coefficient R = 0.81, *p* < 2.2 × 10^−^^16^ between age‐standardized prevalence rate of IBD and GWAS; R = 0.79, *p* < 2.2 × 10^−^^16^ between age‐standardized incidence rate of IBD and GWAS, Figure S8). Notably, even among the 18 correlated clumps, the lead SNPs identified in our bQTL analysis did not reach genome‐wide significance (*p* < 5 × 10^−8^) in the conventional GWAS, indicating the novel genetic variants specifically associated with disease burden. It demonstrated that the bQTL analysis could identify novel loci influencing IBD burden at the population level, which may be missed by conventional GWAS due to population structure differences or the fundamental distinction between individual susceptibility and population‐level burden.

In addition, we found several significant locus (*p* < 1 × 10^−8^) in conventional GWAS meta‐analysis showed a secondary significance (*p* < 1 × 10^−5^) in the bQTLs of IBD, such as rs9891174 (chr17:39875549:T>A, *P_GWAS_* = 1.12 × 10^−17^, *P_prevalence_* = 4.85 × 10^−7^, *P_incidence_* = 1.24 × 10^−6^), and rs2509735 (chr11:76562104:G>A, *P_GWAS_* = 8.71 × 10^−10^, *P_mortality_* = 8.67 × 10^−6^, *P_DALY_* = 3.68 × 10^−6^).

### 2.6 User‐Friendly Web Tool Provides Robust Capabilities for Investigating bQTLs of IBD

To enable a thorough investigation of the bQTLs of IBD, we created an interactive web‐based tool (Figures S9–S13) that offers user‐friendly access to comprehensive summary statistics. The web‐based platform comprises four dedicated modules cataloging lead SNPs associated with age‐standardized prevalence rate, age‐standardized incidence rate, age‐standardized mortality rate, and age‐standardized DALY rate of IBD. Each entry includes detailed annotations, namely SNP ID, beta_CAF_, *p*‐value, chromosome, position, reference/alternative allele, rs identifiers, and credible/causal SNP classification. This integrated resource (available at http://www.onethird‐lab.com/BurdenGenome/IBD) provides a foundational dataset for investigating genetic determinants of IBD's global disease burden.

## 3 Methods

### 3.1 Data Acquisition and Preprocessing

We mapped 1000 Genomes Project genotype data with countries for which Global Burden of Disease (GBD) data were available. We utilized Phase 3 data from the 1000 Genomes Project, which provides a broad representation of human genetic variation and includes 2504 unrelated individuals from 26 populations. For our analysis, we downloaded the VCF files from the FTP site (https://ftp.ncbi.nlm.nih.gov/1000genomes/ftp/1000G_2504_high_coverage/working/20201028_3202_raw_GT_with_annot/) and extracted SNP genotypes for unrelated individuals. Quality control was performed on SNPs with the following removal criteria: (i) minimum allele frequencies (MAF) < 0.01; (ii) Hardy's Weinberg equilibrium p < 0.001. After filtering, 2 621 978 biallelic SNPs on autosomes from 2414 individuals across 26 populations were retained. All SNPs were aligned to the GRCh38/hg38 reference genome. Accordingly, subsequent analyses, including gene mapping, functional enrichment, and visualization, were based on GRCh38/hg38, ensuring consistency in genome mapping.

To ensure the genetic data accurately reflected the populations experiencing localized disease burden in the GBD dataset, we merged genetically similar sub‐populations to estimate representative national allele frequencies: 1) Han Chinese South (CHS), Han Chinese in Beijing (CHB), and Chinese Dai in Xishuangbanna (CDX) were combined into a single “China” group; 2) Indian Telugu in the UK (ITU) and Gujarati Indians in Houston (GIH) into “India”; and 3) Esan in Nigeria (ESN) and Yoruba in Ibadan, Nigeria (YRI) into “Nigeria”. We mapped the countries to GBD data based on the geographic sampling location and ancestral background of the 1000 Genomes cohorts. African Ancestry in Southwest US (ASW) and Utah Residents with European ancestry (CEU) populations were excluded because they do not correspond to specific GBD country‐level populations. No additional populations were excluded due to population stratification or sample size. Consequently, a total of 20 countries (Barbados, Gambia, Nigeria, Kenya, Sierra Leone, China, Vietnam, Japan, Finland, United Kingdom, Italy, Spain, Pakistan, Bangladesh, India, Sri Lanka, Puerto Rico, Colombia, Peru, and Mexico) with both epidemiological and genetic information were included in our analyses. Subsequently, we calculated the frequency of the reference allele for each SNP in 20 countries.

Data on the global burden of IBD from 1990 to 2021 were obtained from the GBD 2021 Results Database. We extracted four key epidemiological indicators for each country and year, including age‐standardized prevalence rate (ASPR), age‐standardized incidence rate (ASIR), age‐standardized mortality rate (ASMR), and age‐standardized disability‐adjusted life years (DALYs). Specifically, the ASPR (per 100 000 population) represents the proportion of individuals living with IBD in a given year, standardized by population age structure to allow cross‐population comparison [24, 25, 26]; The ASIR (per 100 000 population) was calculated as the number of newly diagnosed IBD cases divided by the population size in each year, also adjusted for age [27, 28, 29]; The ASMR (per 100 000 population) corresponds to the annual number of deaths attributed to IBD divided by the total population size, adjusted for age distribution [30, 31, 32]; Finally, DALYs (per 100 000 population) reflect the total number of healthy life years lost due to IBD, encompassing both years of life lost due to premature mortality (YLLs) and years lived with disability (YLDs) [33, 34, 35]. The four key indicators enable standardized assessment of disease burden across countries.

### 3.2 Burden QTL Analysis

To identify bQTLs with the global burden of IBD, we conducted a linear mixed‐effects model [36, 37] in which the age‐standardized prevalence rate, age‐standardized incidence rate, age‐standardized mortality rate, and age‐standardized DALY rate of IBD served as dependent variables, while the reference allele frequency of SNPs was treated as independent variables. Furthermore, to ensure the robustness of these associations and account for potential test statistic inflation, we adjusted for key confounders, including the socio‐demographic Index (SDI), the interaction of reference allele frequency and SDI, country, and year:

$$\begin{array}{ccc} \text{Burden}∼{\text{SNP}}_{RAF} & + & {\text{Cov}}_{SDI}+{\text{Interaction}}_{\left(SNP\;\times \;SDI\right)}\\ & + & \text{Random effect} \end{array}$$

of which, *Burden* is the global burden of IBD, including age‐standardized prevalence rate, age‐standardized incidence rate, age‐standardized mortality rate, and age‐standardized DALY rate in 20 countries from 1990 to 2021; *SNP_RAF_* is the reference allele frequency of SNPs in the corresponding countries; *Cov_SDI_* is the socio‐demographic index (SDI) across 20 countries each year; *Interaction_(SNP×SDI)_* is the interaction of reference allele frequency and SDI; *Random effect* indicates the year and country accordingly.

Given that the allele frequencies ranged from 0 to 1, we evaluated their disease burden effects per centi‐allele frequency (CAF, 0.01 allele frequency) increase (beta_CAF_). To control the false discovery rate (FDR) across all analyses, we applied a rigorous threshold of *p*‐value less than 1 ×10^−8^ to identify significant associations. While our analysis involved 2 621 978 raw SNPs, the number of effectively independent tests is substantially lower due to extensive linkage disequilibrium (LD). Following established GWAS standards that estimate approximately one million independent common variants in the human genome (corresponding to 5 × 10^−8^), we further adjusted this criterion to account for the four distinct epidemiological indicators tested (5 × 10^−8^/4 = 1.25 × 10^−8^). We rounded this to 1 × 10^−8^ to provide more conservatism.

Subsequently, we performed linkage disequilibrium (LD) clumping to identify lead SNPs across risk loci. The LD coefficient r^2^ for any two SNPs located on non‐homologous chromosomes was calculated. Notably, it was calculated directly from genotype data within each individual 1000 Genomes population, rather than from a pooled sample combining all populations. First, to select independently significant SNPs, we started with the most significant variant (lowest *p*‐value) and excluded all SNPs within a ±500 kb window (1 Mb total) showing high LD (r^2^> 0.6). This step aimed to remove highly redundant variants that represent the same primary association. Second, to define lead SNPs, we repeated the clumping procedure with a more stringent LD threshold (r^2^> 0.1) within the same 1 Mb window. SNPs with r^2^> 0.1 were grouped into clumps, and the variant with the smallest *p*‐value was designated as the lead SNP for each clump. The 1 Mb window is a commonly observed range based on the typical LD decay patterns in the human genome, where LD between sites separated by more than 500 kb is generally negligible. The LD threshold (r^2^> 0.1) is widely used in large‐scale GWAS to balance the capture of distinct variants while ensuring their statistical independence [38, 39]. All reported P‐values and effect sizes were derived from the initial association analysis (pre‐clumping).

### 3.3 Enrichment Analysis

We employed the clusterProfiler package [40] to annotate gene ontology (GO) terms, including biological processes (GO:BP), cellular compartments (GO:CC), and molecular function (GO:MF). Given the potential redundancy in the resulting list of GO terms, where both parent and child terms may appear significant, we applied the simplify function (cutoff = 0.7) to reduce term overlap and retain the most significant entries. To further elucidate biological pathways, we conducted Reactome and KEGG pathway enrichment analysis with the ReactomePA [41] and clusterProfiler packages, respectively. All statistical significance thresholds were adjusted for multiple testing using the Benjamini–Hochberg (BH) method to control the false discovery rate.

### 3.4 Fine‐Mapping Analysis

For each lead SNP, we extracted all nearby variants as a clump with r^2^ > 0.1 and a region of ± 250 kb. Within each clump, we employed approximate Bayesian factors (ABF) of SNP to identify credible causal variants in this region. The 99% credible set for each lead SNP was determined by ranking all SNPs (the clump of the lead SNP) according to their posterior probabilities and then including ranked SNPs until their cumulative posterior probabilities reached or exceeded 0.99. The putative causal variants were determined as SNPs with the highest posterior probabilities.

### 3.5 Colocalization Analysis

We performed colocalization between bQTLs of IBD and genetic associations identified by traditional GWAS. GWAS summary statistics for IBD were obtained from the IEU OpenGWAS project, which was based on GRCh37/hg19. To ensure precise correspondence during colocalization, we used dbSNP Reference Identifiers (rsIDs) as the universal keys to map the GWAS variants to their corresponding genomic coordinates, including chromosome number and position in GRCh38, to ensure the consistency and accuracy of the results. This study comprised 12 716 084 SNPs and involved 12 882 cases and 21 770 controls. The correlation of significance patterns was measured by Pearson correlation. Significant correlations were defined by R greater than 0.5 and *p*‐value less than 0.05.

## 4 Discussion

To explore whether genetic factors account for the burden of IBD across diverse countries, we present the large‐scale burden QTL analysis by combining the epidemiological information of IBD from 1990 to 2021 in the GBD study with 2 621 978 SNPs in the 1000 Genomes Project across 20 matched countries.

We identified 774 lead variants with age‐standardized prevalence rates, 611 with age‐standardized incidence rates, 56 with age‐standardized mortality rates, and 18 with age‐standardized DALY rates. The allele frequency distributions of lead variants across 20 countries were presented in Tables S8–S11 (Figures S14–S17). Among them, 385 demonstrated significance in two metrics (384 for both age‐standardized prevalence rate and age‐standardized incidence rate; 1 for both age‐standardized incidence rate and age‐standardized DALY rate). Subsequently, we identified 800 putative causal variants for the burden of IBD, which showed distinct genomic hotspots: chromosome 3 harbored the highest number of putative causal variants for age‐standardized prevalence rate, followed by chromosomes 1 and 4, while chromosomes 1, 2, 4, and 11 contained the highest density of putative causal variants for age‐standardized incidence rate. The putative causal SNPs of age‐standardized mortality rate of IBD showed the highest density on chromosomes 8, 1, and 2, while the age‐standardized DALY rate analysis revealed the fewest putative causal SNPs, primarily localized on chromosome 4 (Figure S7). We observed that some putative causal variants were located within genes related to immunoglobulin, inflammatory response, and intestinal cancers. Intriguingly, we found other putative causal variants located in neuronal‐related protein‐coding genes. For example, *ASTN1* (*astrotactin 1*, rs12722726 [chr1:177102597:G>A], *P_prevalence_ =* 4.94 × 10^−11^) is a neuronal adhesion molecule required for glial‐guided migration of young postmitotic neuroblasts in cortical regions of the developing brain [https://www.ncbi.nlm.nih.gov/gene/460]. *KCNN3* (*potassium calcium‐activated channel subfamily N member 3*, rs10494301 [chr1:154728742:T>C], *P_incidence_ =* 9.67 × 10^−9^) encodes an integral membrane protein that forms a voltage‐independent calcium‐activated channel, which is thought to regulate neuronal excitability by contributing to the slow component of synaptic afterhyperpolarization [https://www.ncbi.nlm.nih.gov/gene/3782]. *MYT1* (*myelin transcription factor 1*, rs6122250 [chr20:64125169:G>A], *P_mortality_ =* 6.99 × 10^−9^) encoded a member of a family of neural‐specific, zinc finger‐containing DNA‐binding proteins [https://www.ncbi.nlm.nih.gov/gene/4661]. The probable reason is that the gastrointestinal tract is densely innervated by a highly organized neuronal architecture, coordinating vital physiological functions [42, 43, 44, 45, 46, 47]. The impact of neuro‐related factors on the disease burden of IBD still needs to be further explored.

In this study, we first integrated epidemiological information over 32 years and genotype data from over 2.6 million SNPs to delineate the influence of population genetic architecture on the global burden of IBD, complementing conventional GWAS that focus on individual‐level genetic susceptibility. For the bQTL analysis, we adjusted for socioeconomic and environmental confounders by introducing SDI as a covariate and corresponding country and year as random effects in a mixed‐effects model. In addition, considering the interaction of genetics and environment, we further adjusted for the interaction term (SNP × SDI). This minimized the impact of population stratification or temporal trends and ensured that the identified bQTL reflected the impact of genetic information on the burden of IBD. However, due to the limited availability of complete data across different regions, the adjustments could not fully rule out the potential environmental heterogeneities, such as specific westernized diet scores or localized urbanization processes. Future studies should aim to integrate more comprehensive environmental datasets across diverse populations to validate and refine these findings. Nevertheless, our results demonstrated that although often attributed to socioeconomic factors, the heredity of IBD contributed a substantial portion to disease burden across populations. Epidemiological transition models must therefore incorporate both environmental indicators and molecular genetic determinants to achieve a comprehensive understanding of the global burden of IBD.

Recent large‐scale cross‐population genome‐wide association analysis studies [48, 49] reported a total of nine IBD‐associated SNPs. There were two overlaps with the variants included in our study, but neither showed significant associations in our bQTL analyses. The discrepant findings further underscore the necessity and novelty of our study. Traditional GWAS are designed to identify variants associated with the individual risk of IBD. In contrast, we integrated population‐level allele frequencies with country‐specific IBD burden metrics, rather than testing genotype‐phenotype associations at the individual level. Our bQTL analysis identifies variants associated with the long‐term epidemiological burden (prevalence, incidence, mortality, and DALYs). It revealed genetic association with the burden of IBD across different national contexts rather than individual susceptibility. Therefore, the lack of replication of these specific SNPs further highlights the value of our analysis. It enables capturing global population‐level genetic association with IBD burden, which has not been systematically conducted in previous GWAS.

Nevertheless, this study has some limitations that should be acknowledged. First, there are 20 countries that can simultaneously obtain epidemiological information and genotype data. These countries are unevenly distributed across continents, with a higher representation of European and Asian populations compared to African and Oceanian regions. While the 1000 Genomes Project provides a robust starting point, this geographic imbalance may limit the generalizability of our findings to the global population. We conducted a leave‐one‐country‐out sensitivity analysis to test the robustness of our estimates to country selection. The mixed‐effects model was iteratively re‐estimated 20 times, with one country excluded in each iteration. As shown in Figure S19, the beta_CAF_ of the top 5 lead SNPs of ASPR remained remarkably stable across all subsets, demonstrating that our global estimates are not influenced by any single country. For instance, for rs7633471, generated beta_CAF_ estimates ranged from −10.54 to −8.63 (Table S2, compared to the 20‐country estimate of −10.00), with all values remaining negative and statistically significant (*p* < 1 × 10^−8^). The largest relative change was observed when excluding Finland (from −10.00 to −8.63), but the magnitude of this change was modest relative to the standard error of the estimate (SE_CAF_ = 0.96, change 1.37 ≈ 1.4 standard errors). These results confirm that our findings are not driven by any single country and are robust to the exclusion of individual countries. Future research should prioritize expanding geographic coverage by integrating localized biobank data and more diverse genomic resources to validate and extend these findings. Second, IBD includes two distinct subtypes, Crohn's disease and ulcerative colitis, and each with potentially unique genetic contributions to disease burden. Due to the lack of subtype‐specific epidemiological data in the GBD, we analyzed IBD as a composite entity. While this approach enabled us to capture general genetic associations with global IBD burden, it may obscure variants that specifically impact Crohn's disease or ulcerative colitis, as well as potential interactions with environmental and socioeconomic factors that differ between subtypes. Future studies incorporating detailed, subtype‐specific epidemiological and genetic data would allow for a more precise delineation of subtype‐specific bQTLs and their contributions to IBD burden. Such analyses would facilitate the identification of subtype‐specific bQTLs, improve our understanding of the underlying biological mechanisms of IBD, and support the development of precision public health strategies. Thirdly, while the 1000 Genomes Project provides high‐quality reference data, some included populations are represented by relatively small sample sizes (e.g., *n* < 100). The standard error of allele frequency estimates decreases with the number of alleles (twice the sample size, two alleles per individual) and is relatively small for common variants. It is acceptable for regression analyses aimed at detecting cross‐country correlations. ChinaMAP (http://chinamapwgs.mbiobank.com/) and IndiGenomes (http://clingen.igib.res.in/indigen/) provide whole‐genome sequencing data from 10 588 Chinese individuals and 1029 Indian individuals, respectively. We retrieved allele frequencies for our bQTLs from IndiGenomes and ChinaMAP, and recalculated effect sizes and *p*‐values after replacing the India and China 1000G frequencies with these new values. There were 731/748 (97.73%) ASPR lead SNPs, 569/585 (97.26%) ASIR lead SNPs, 46/52 (88.46%) ASMR SNPs, and 18/18 (100%) ASDR lead SNPs remaining significant at *p* < 1×10^−8^. The beta_CAF_ are also highly consistent with the results from 1000 Genomes (Table S3). The results indicate that our bQTLs are not a reflection of sampling bias inherent to the 1000 Genomes sample size. However, smaller sample sizes may not fully capture the fine‐scale genetic structures. Notably, allele frequencies from 1000 Genomes samples are widely used as proxies for population‐level genetic backgrounds rather than precise national estimates. Future studies with expanded reference samples are needed to validate and extend our findings. Finally, we would like to clarify that the putative causal variants were identified without functional experimental validation, such as luciferase assays or CRISPR. Because our study is a large‐scale population‐level genomics analysis involving 800 loci across four burden metrics, high‐throughput experimental validation of every candidate is currently beyond the scope and resource of this work. Future functional experiments and independent replication will be essential to confirm the biological relevance of the loci we have prioritized. In addition, our colocalization approach differs from traditional Bayesian methods. Because our bQTL framework is based on population‐level allele frequency differences rather than individual‐level GWAS summary statistics, conventional Bayesian colocalization approaches cannot be directly applied without additional assumptions. Therefore, our analysis should be interpreted as evaluating regional genetic concordance rather than demonstrating shared causal variants.

In conclusion, our study revealed genetic association with the global burden of IBD, contributing to formulating targeted strategies that reduce the burden of IBD.

## Author Contributions

Y.S.J. conceived and contributed to the work. C.S., S.Y.W., J.X.T., H.Y.C., C.Y., J.C.W, J.X., L.D., Y.B.Z., Y.P.Z., R.L.L., L.N.Y., W.S, S.L.L., N.W., Q.D.R., Y.G., Y.X., C.W., H.S.T., C.Z., Y.D., G.P.T., Z.W.S, Y.S.J. W.H.L., M.M.Z., and H.C.L. drafted and modified the manuscript.

## Funding

This work was supported by the National Natural Science Foundation of China Grant Nos. 31970651, 92046018; the Excellent Youth Support Plan of the Education Department of Heilongjiang Province Grant No. YQJH2023036; Marshal Initiative Funding Grant No. HMUMIF‐22010; XingLian Outstanding Talent Support Program 2024; Zhejiang Provincial Natural Science Foundation of China Grant No. ZCLMS25H0402.

## Conflicts of Interest

The authors declare no conflicts of interest.

## Supporting information




**Supporting File 1**: advs77646‐sup‐0001‐SuppMat.docx.

**Supporting File 2**: advs77646‐sup‐0002‐Table S1–S11.xlsx.

## Data Availability

Data are available in a public, open‐access repository. We downloaded the epidemiological data of IBD from the Global Health Data Exchange (GHDx) query tool (https://vizhub.healthdata.org/gbd‐results/). The raw variant data in VCF format were obtained from the 1000 Genomes Project (https://ftp.ncbi.nlm.nih.gov/1000genomes/ftp/1000G_2504_high_coverage/working/20201028_3202_raw_GT_with_annot/).
