# Supplementary material for: Integrating Genetics With Epidemiological Measurements Identifies Burden QTLs of Inflammatory Bowel Disease across 20 Countries
Source: Adv Sci (Weinh). 2026 Sep 27:e19007. Online ahead of print. doi: 10.1002/advs.202519007 (PMC13616370; doi:10.1002/advs.202519007)
Supplement: Supplementary file 1 [file ADVS-9999-e19007-s002.docx]

**Supplementary Materials for**

**Integrating genetics with epidemiological measurements identifies Burden QTLs of Inflammatory Bowel Disease across 20 countries**

# Chen Sun^1,†^, Siyu Wei^1,†^, Junxian Tao^1,†^, Haiyan Chen^1,†^, Chi Yan^1^, Jiacheng Wang^1^, Jing Xu^2^, Lian Duan^3^, Yuanbo Zhan^4^, Yuping Zou^1^, Ruilin Li^1^, Linna Yuan^1^, Wei She^1^, Songlin Lu^1^, Ning Wang^1^, Qinduo Ren^1^, Yan Guo^1^, Yuan Xu^1^, Chang Wang^1^, Hongsheng Tian^1^, Chen Zhang^5^, Yu Dong^5^, Guoping Tang^6^, Zhenwei Shang^1^, Yongshuai Jiang^1,*^, Wenhua Lv^1,*^, Mingming Zhang^1,*^, Hongchao Lyu^1,*^,

^1^College of Bioinformatics Science and Technology, Harbin Medical University, Harbin, China.

^2^Faculty of Information Engineering and Automation, Kunming University of Science and Technology, Kunming, China.

^3^Central Laboratory, The First Affiliated Hospital of Wenzhou Medical University, Wenzhou, China

^4^Department of Periodontology and Oral Mucosa, The Second Affiliated Hospital of Harbin Medical University, Harbin, China.

^5^Harbin Medical University, Harbin, China.

^6^The Fourth Affiliated Hospital, Zhejiang University School of Medicine, Yiwu, China.

† These authors contributed equally to this work.

* Correspondence:

Hongchao Lyu, College of Bioinformatics Science and Technology, Harbin Medical University, 194 Xuefu Road, Nangang District, Harbin, Heilongjiang Province, China. E-mail: hongchaolyu@hrbmu.edu.cn.

Mingming Zhang, College of Bioinformatics Science and Technology, Harbin Medical University, 194 Xuefu Road, Nangang District, Harbin, Heilongjiang Province, China. E-mail: zhangmingming@ems.hrbmu.edu.cn.

Wenhua Lv, College of Bioinformatics Science and Technology, Harbin Medical University, 194 Xuefu Road, Nangang District, Harbin, Heilongjiang Province, China. E-mail: wenhua.1989.happy@163.com.

Yongshuai Jiang, College of Bioinformatics Science and Technology, Harbin Medical University, 194 Xuefu Road, Nangang District, Harbin, 150081, Heilongjiang Province, China. phone numbers: +86 13258669501; E-mail: [jiangyongshuai@gmail.com.](mailto:jiangyongshuai@gmail.com;)

###### 1. Global trend of IBD burden from 1990 to 2021.

The Global Burden of Disease (GBD) 2021 study employed multiple approaches to integrate diverse data sources while accounting for disease-specific epidemiological patterns. The primary analytical framework for disease impact estimation was Bayesian meta-regression. Our research specifically collected data on four key age-standardized metrics for inflammatory bowel disease (IBD): prevalence rate, incidence rate, mortality rate, and Disability-Adjusted Life Years (DALYs) rate. From these datasets, we extracted critical variables including year and geographic location. The incidence rate was calculated as new cases per 100,000 population, while mortality rate represented annual deaths per 100,000 population. DALYs quantified healthy life years lost annually per 100,000 people, enabling standardized burden comparisons across regions.

To assess the collective IBD burden across our 20 study countries, we incorporated demographic data to calculate population-weighted estimates. For each year, we first determined each country's population proportion relative to the total population of all 20 nations. We then computed weighted averages of disease burden indicators (including all four age-standardized rates) to represent the overall burden, with population proportions serving as weights. This approach generated comprehensive global age-standardized rates through summation across all countries. We employed weighted Monte Carlo simulations to estimate 95% uncertainty intervals (95% UIs). Under the assumption of normally distributed uncertainty in the source data, we derived Standard Errors by dividing the uncertainty interval width by 3.92 (2 × 1.96). Our Monte Carlo simulation generated a random sample matrix with normally distributed values, using the observed burden metrics as means and the calculated Standard Errors as standard deviations. For each of the 10,000 simulations (represented as matrix columns), we calculated weighted sums to produce simulated age-standardized rate (ASR) values. The 95% UIs were then determined from the 2.5th and 97.5th percentiles of these simulated ASR values. Finally, we estimated percentage changes (expressed as estimated annual percentage changes, EAPCs) for all four age-standardized metrics between 1990 and 2021 to analyze temporal trends in IBD burden.

**Table S1. Age-standardized rates (per 100,000 population) of prevalence, incidence, mortality, and disability-adjusted life years (DALYs) attributable to inflammatory bowel disease in 20 countries.**

|  | **Prevalence** | **Incidence** | **Death** | **DALYs** |
| --- | --- | --- | --- | --- |
| 1990 | 25.62[23.34,27.85] | 2.91[2.66,3.17] | 0.59[0.48,0.69] | 18.59[16.03,21.11] |
| 1991 | 25.91[23.67,28.08] | 2.95[2.69,3.21] | 0.58[0.48,0.68] | 18.58[16.01,21.10] |
| 1992 | 26.21[23.96,28.44] | 2.99[2.72,3.25] | 0.58[0.48,0.67] | 18.60[16.16,21.04] |
| 1993 | 26.49[24.20,28.77] | 3.03[2.76,3.30] | 0.57[0.47,0.67] | 18.44[15.99,20.93] |
| 1994 | 26.75[24.46,29.05] | 3.07[2.79,3.34] | 0.56[0.47,0.66] | 18.29[15.96,20.58] |
| 1995 | 26.97[24.64,29.01] | 3.11[2.83,3.39] | 0.56[0.48,0.66] | 18.23[15.95,20.52] |
| 1996 | 27.23[24.89,29.58] | 3.16[2.87,3.44] | 0.55[0.46,0.64] | 17.95[15.69,20.18] |
| 1997 | 27.56[25.14,29.99] | 3.23[2.93,3.52] | 0.54[0.46,0.62] | 17.72[15.53,19.84] |
| 1998 | 27.91[25.44,30.38] | 3.30[3.00,3.61] | 0.54[0.45,0.63] | 17.69[15.54,19.88] |
| 1999 | 28.23[25.65,30.84] | 3.37[3.05,3.67] | 0.53[0.45,0.61] | 17.59[15.46,19.70] |
| 2000 | 28.45[25.67,31.10] | 3.41[3.10,3.72] | 0.53[0.45,0.62] | 17.56[15.34,19.67] |
| 2001 | 28.59[25.92,31.21] | 3.43[3.12,3.74] | 0.54[0.45,0.62] | 17.55[15.42,19.65] |
| 2002 | 28.71[26.06,31.39] | 3.43[3.13,3.74] | 0.53[0.45,0.62] | 17.43[15.31,19.50] |
| 2003 | 28.85[26.26,31.43] | 3.44[3.13,3.75] | 0.54[0.46,0.62] | 17.41[15.29,19.59] |
| 2004 | 29.03[26.48,31.57] | 3.45[3.14,3.76] | 0.54[0.46,0.62] | 17.38[15.30,19.47] |
| 2005 | 29.28[26.65,31.83] | 3.47[3.16,3.79] | 0.53[0.45,0.60] | 17.06[15.12,19.02] |
| 2006 | 29.78[27.07,32.45] | 3.53[3.22,3.85] | 0.50[0.44,0.57] | 16.68[14.81,18.50] |
| 2007 | 30.55[27.83,33.24] | 3.63[3.30,3.97] | 0.49[0.43,0.55] | 16.44[14.74,18.16] |
| 2008 | 31.40[28.57,34.33] | 3.75[3.41,4.09] | 0.47[0.42,0.53] | 16.28[14.61,17.97] |
| 2009 | 32.15[29.11,35.17] | 3.84[3.49,4.20] | 0.46[0.41,0.51] | 16.14[14.56,17.70] |
| 2010 | 32.61[29.61,35.73] | 3.90[3.55,4.26] | 0.45[0.40,0.50] | 15.94[14.36,17.53] |
| 2011 | 32.79[29.78,35.85] | 3.92[3.56,4.28] | 0.44[0.39,0.48] | 15.73[14.12,17.34] |
| 2012 | 32.86[29.74,35.94] | 3.93[3.57,4.30] | 0.42[0.38,0.47] | 15.44[13.95,16.95] |
| 2013 | 32.85[29.73,36.00] | 3.93[3.57,4.30] | 0.42[0.37,0.46] | 15.25[13.73,16.77] |
| 2014 | 32.80[29.60,35.99] | 3.93[3.56,4.30] | 0.41[0.37,0.46] | 15.00[13.40,16.56] |
| 2015 | 32.74[29.59,35.93] | 3.94[3.56,4.32] | 0.40[0.35,0.45] | 14.72[13.11,16.34] |
| 2016 | 32.41[29.23,35.58] | 3.91[3.54,4.28] | 0.39[0.34,0.44] | 14.54[12.96,16.17] |
| 2017 | 32.76[28.77,34.88] | 3.85[3.49,4.22] | 0.39[0.33,0.44] | 14.36[12.70,16.03] |
| 2018 | 31.11[28.01,34.21] | 3.79[3.43,4.14] | 0.38[0.33,0.44] | 14.11[12.46,15.77] |
| 2019 | 30.80[27.73,33.80] | 3.77[3.41,4.13] | 0.38[0.32,0.43] | 13.90[12.22,15.56] |
| 2020 | 30.92[27.88,33.88] | 3.80[3.45,4.16] | 0.37[0.32,0.43] | 13.66[12.04,15.25] |
| 2021 | 30.82[27.74,33.81] | 3.80[3.44,4.17] | 0.37[0.31,0.42] | 13.60[11.92,15.27] |
| Percent change between 1990 and 2021 | 20.30% | 30.58% | -37.29% | -26.84% |

**Supplementary Fig. 1 (A-D) Top 5 bQTLs about age-standardized prevalence rate of IBD. The Y-axis presents the independent genetic effects of SNPs on IBD burden after adjusting for SDI and its interaction terms (****). ASPR: age-standardized prevalence rate.**

**Supplementary Fig. 2 (A-D) Top 5 bQTLs about age-standardized incidence rate of IBD. The Y-axis presents the independent genetic effects of SNPs on IBD burden after adjusting for SDI and its interaction terms (****). ASIR: age-standardized incidence rate.**

**Supplementary Fig. 3 (A-D) Top 5 bQTLs about age-standardized mortality rate of IBD. The Y-axis presents the independent genetic effects of SNPs on IBD burden after adjusting for SDI and its interaction terms (****). ASMR: age-standardized mortality rate.**

**Supplementary Fig. 4 (A-D) Top 5 bQTLs about age-standardized DALY rate of IBD. The Y-axis presents the independent genetic effects of SNPs on IBD burden after adjusting for SDI and its interaction terms (****). ASDR: age-standardized DALY rate.**

**Supplementary Fig. 5 Venn plot for lead SNPs among age-standardized prevalence, incidence, mortality, DALYs rate.**

**Supplementary Fig. 6 Venn plot for causal SNPs among age-standardized prevalence, incidence, mortality, DALYs rate.**

**Supplementary Fig. 7 The density of putative causal SNPs of age-standardized prevalence rate, age-standardized incidence rate, age-standardized mortality rate, and age-standardized DALYs rate on each chromosome.**

**Supplementary Fig. 8 Colocalization of the clump of lead SNP rs7608429 (chr2:6835704). ASPR: age-standardized prevalence rate; ASIR: age-standardized incidence rate.**

**Supplementary Fig. 9 Homepage of the website.**

**Supplementary Fig. 10 About page of the website.**

**Supplementary Fig. 11 Result page of the website.**

**Supplementary Fig. 12 Quick Search on the result page of the website.**

**Supplementary Fig. 13 Contact page of the website.**

**Supplementary Fig. 14 Allele frequency distributions of top 50 lead variants for ASPR in IBD.**

**Supplementary Fig. 15 Allele frequency distributions of top 50 lead variants for ASIR in IBD.**

**Supplementary Fig. 16 Allele frequency distributions of lead variants for ASMR in IBD.**

**Supplementary Fig. 17 Allele frequency distributions of lead variants for DALYs in IBD.**

**Supplementary Fig. 18 Biological functions of 8 pleiotropic genes simultaneously influencing prevalence and incidence and overlapping with previously reported IBD-associated genes.**

**Supplementary Fig. 19 Sensitivity analysis of the top 5 lead variants for ASPR in IBD by sequential exclusion of each of the 20 countries. The X-axis represents the countries excluded each time, and the Y-axis represents the effect size (beta_CAF_).**

**Table S2. Sensitivity analysis of the lead SNP rs7633471 by sequential exclusion of each of the 20 countries.**

| **exclude_country** | **Beta_CAF_20_countries** | **Beta_CAF_19_countries** | **change** |
| --- | --- | --- | --- |
| Sri Lanka | -10.0028 | -9.9983 | 0.0045 |
| Spain | -10.0028 | -9.9896 | 0.0132 |
| Sierra Leone | -10.0028 | -9.9900 | 0.0128 |
| Pakistan | -10.0028 | -10.0145 | -0.0117 |
| Bangladesh | -10.0028 | -9.9849 | 0.0179 |
| Viet Nam | -10.0028 | -9.9815 | 0.0213 |
| Nigeria | -10.0028 | -9.9865 | 0.0163 |
| Colombia | -10.0028 | -10.0193 | -0.0165 |
| China | -10.0028 | -9.9851 | 0.0176 |
| Kenya | -10.0028 | -9.9738 | 0.0290 |
| Barbados | -10.0028 | -10.0544 | -0.0516 |
| United Kingdom | -10.0028 | -10.0675 | -0.0674 |
| Mexico | -10.0028 | -9.9460 | 0.0568 |
| Puerto Rico | -10.0028 | -10.0968 | -0.0940 |
| Japan | -10.0028 | -10.1706 | -0.1678 |
| Gambia | -10.0028 | -10.4711 | -0.4683 |
| Peru | -10.0028 | -10.4869 | -0.4841 |
| India | -10.0028 | -10.5419 | -0.5391 |
| Italy | -10.0028 | -9.2472 | 0.7556 |
| Finland | -10.0028 | -8.6284 | 1.3744 |

**Table S3. Effect sizes of top 5 lead SNPs for ASPR, ASIR, ASMP, and ASDR of IBD in IndiGenomes and ChinaMAP.**

| **Measure** | **SNP** | **1000G** | | **IndiGenomes and ChinaMAP** | |
| --- | --- | --- | --- | --- | --- |
|  |  | $\text{β}_{\text{CAF}}$ | **P** | $\text{β}_{\text{CAF}}$ | **P** |
| ASPR | rs7633471 | -10.00 | 2.59e-19 | -10.53 | 2.50e-20 |
|  | rs35759242 | 9.01 | 1.42e-16 | 9.05 | 2.03e-16 |
|  | rs1498523 | -8.02 | 1.52e-15 | -7.72 | 1.81e-12 |
|  | rs7494925 | -10.11 | 1.92e-15 | -10.17 | 2.37e-15 |
|  | rs6800743 | -9.01 | 6.46e-15 | -8.88 | 4.79e-15 |
| ASIR | rs7633471 | -1.02 | 5.62e-20 | -1.07 | 8.28e-20 |
|  | rs315502 | 0.77 | 5.99e-17 | 0.76 | 1.42e-15 |
|  | rs7763927 | 0.87 | 1.43e-16 | 0.85 | 8.53e-14 |
|  | rs6800743 | -0.87 | 1.01e-15 | -0.86 | 1.77e-16 |
|  | rs17069646 | 1.18 | 1.02e-15 | 1.12 | 4.86e-15 |
| ASMR | rs11674520 | -0.14 | 6.60e-13 | -0.14 | 8.82e-13 |
|  | rs9577763 | -0.12 | 1.49e-11 | -0.12 | 1.06e-11 |
|  | rs16920712 | -0.15 | 1.77e-11 | -0.18 | 3.17e-13 |
|  | rs11774172 | 0.13 | 7.35e-11 | 0.15 | 8.54e-13 |
|  | rs59308021 | -0.13 | 8.18e-11 | -0.12 | 6.38e-10 |
| ASDR | rs189090665 | -3.83 | 6.74e-11 | -3.87 | 5.00e-11 |
|  | rs3847282 | 1.76 | 7.62e-11 | 1.75 | 8.91e-11 |
|  | rs1959196 | 3.31 | 5.92e-10 | 3.35 | 4.52e-10 |
|  | rs139193 | -1.67 | 8.76e-10 | -1.69 | 6.44e-10 |
|  | rs11156650 | 2.91 | 1.53e-09 | 2.94 | 1.25e-09 |
